# Dietary and Lifestyle Risk Factors in Primary Sclerosing Cholangitis: In Search of Mechanistic Explanations and Health Improvement

**DOI:** 10.1016/j.tjnut.2025.10.041

**Published:** 2025-10-31

**Authors:** Ruslan A Mammadov, Henk P Roest, Luc JW van der Laan, Maikel P Peppelenbosch

**Affiliations:** 1 Department of Surgery, Erasmus MC Transplant Institute, University Medical Center Rotterdam, Rotterdam, The Netherlands; 2 Department of Gastroenterology and Hepatology, Erasmus University Medical Center Rotterdam, Rotterdam, The Netherlands

**Keywords:** primary sclerosing cholangitis, bile acid metabolism, gut microbiota, short-chain fatty acids, immune modulation, dietary interventions

## Abstract

Primary sclerosing cholangitis (PSC) is a chronic, immune-mediated liver disease characterized by progressive inflammation and fibrosis of the bile ducts. Although much of the research has focused on genetic and autoimmune mechanisms, growing interest has emerged in the role of modifiable risk factors, including dietary and lifestyle factors, in the pathogenesis and progression of PSC. This review aims to provide a comprehensive synthesis of the current literature on dietary and lifestyle influences on PSC, with particular attention to underlying molecular pathways. Key factors discussed include smoking, alcohol consumption, physical activity, and medical history factors such as appendectomy, tonsillectomy, and hormonal therapies. Additionally, the review explores the impact of dietary components, such as coffee, vitamins, probiotics, and dietary fibers, on PSC progression. Potential mechanisms highlighted include alterations in bile acid metabolism, gut microbiota dysbiosis, disruption of intestinal barrier integrity, and modulation of innate and adaptive immune responses through cytokine signaling and pattern recognition receptor pathways. Emerging evidence also suggests that coffee constituents may influence oxidative stress pathways and fibrogenesis, whereas dietary fibers and probiotics may act through short-chain fatty acid production and anti-inflammatory signaling. Although the evidence for many of these factors remains inconclusive, these molecular insights point to diet-induced immunometabolism modulation as a promising area for therapeutic intervention. Further prospective studies and randomized trials are needed to clarify these mechanisms and develop targeted strategies to modify disease onset and progression.

## Graphical abstract

**Figure:**
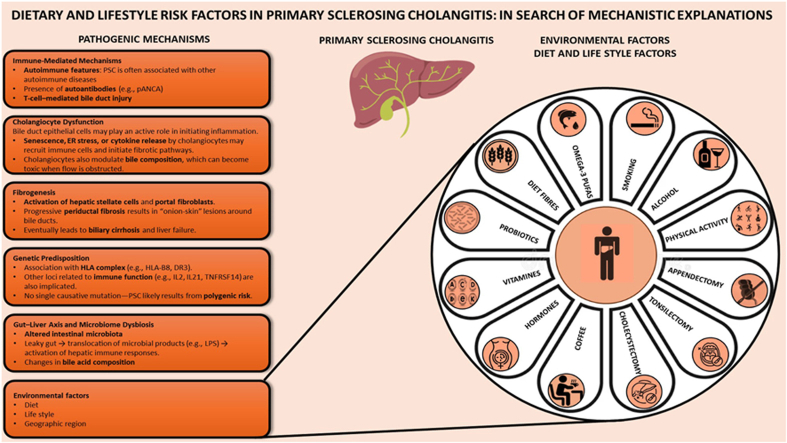

## Introduction

Primary sclerosing cholangitis (PSC) is a chronic, immune-mediated liver disease characterized by progressive inflammation and fibrosis of the intrahepatic and extrahepatic bile ducts. Although it is a rare condition, its impact is significant due to the high risk of cirrhosis, liver failure, and hepatobiliary malignancies, including cholangiocarcinoma [1]. PSC has a strong and well-documented association with inflammatory bowel disease (IBD), particularly ulcerative colitis (UC), yet its precise etiology remains elusive [2]. Several mechanisms have been proposed to explain this link. One hypothesis involves aberrant gut–liver immune interactions, where increased intestinal permeability allows translocation of bacterial products or antigens to the portal circulation, triggering immune-mediated cholangiopathy [3]. Another proposed mechanism centers on molecular mimicry, in which immune responses to gut microbiota cross-react with bile duct antigens. Additionally, genetic susceptibility, particularly variants in human leukocyte antigen and immune-regulatory genes, may predispose individuals to both conditions [4]. Dysbiosis and altered bile acid metabolism have also been implicated, suggesting a multifactorial pathogenesis involving environmental and immunogenetic factors [5].

Clinical presentation is frequently silent, with many cases detected through abnormal liver tests, though symptoms such as fatigue, pruritus, or cholangitis can occur [[6], [7], [8]]. No medical therapy has been proven to halt disease progression; patient management focuses on surveillance and symptom control, with liver transplantation as the only curative option [6,7]. To date, most research has focused on genetic susceptibility, autoimmune mechanisms, and the role of the gut microbiome [3,8]. The lack of mechanistic insight into pathophysiology precludes targeted treatment, focusing attention on less specific approaches potentially counteracting the PSC disease process.

Hence, interest has emerged in potentially modifiable factors, especially dietary habits, lifestyle behaviors, and medical history elements, that may influence the development or progression of PSC. These factors include smoking status, alcohol intake, prior appendectomy, use of hormonal therapies, and exposure to various dietary components such as coffee, vitamins, and probiotics. Although surgical and medical history are briefly reviewed for context, the primary focus of this review is on modifiable lifestyle and nutritional factors that may influence the onset, progression, or phenotype of PSC.

Understanding the possible influence of these factors on the natural history of PSC is essential for several reasons. First, identifying modifiable risk contributors may offer insights into PSC pathogenesis beyond the existing amount of genetic, immunological, and molecular studies, potentially allowing the rational design of novel therapeutic avenues directly interfering with the disease process. Second, such knowledge can inform preventive strategies and lifestyle counseling in high-risk populations, including those with IBD. Third, if understanding these factors sheds novel mechanistic light on the pathogenesis of PSC, the knowledge might be translatable to other, similar pathologies which elude current medical science, for example, primary biliary cirrhosis or autoimmune hepatitis.

Thus prompted, in this review, we present a comprehensive synthesis of the current literature on dietary and lifestyle risk factors associated with PSC, focusing on their potential link to molecular mechanisms. We aim to critically evaluate the strength and consistency of existing evidence in this light and identify knowledge gaps that warrant further investigation.

## Lifestyle and Behavioral Risk Factors

### Smoking

**FIGURE 1:**
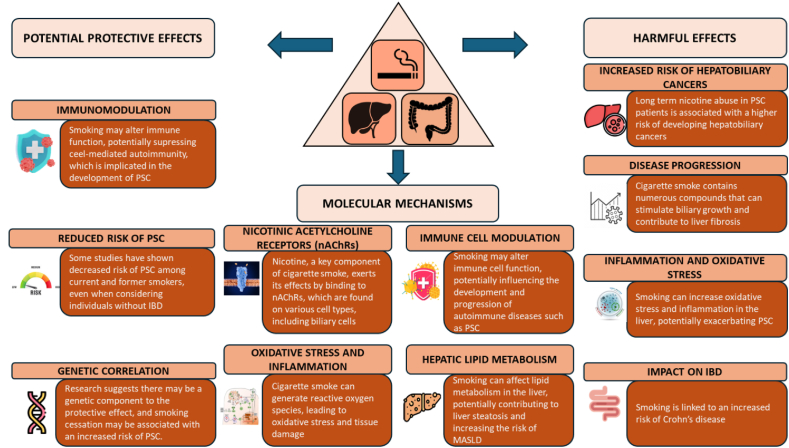
Hypothesized mechanisms linking smoking to primary sclerosing cholangitis (PSC). IBD, inflammatory bowel disease; MASLD, metabolic dysfunction–associated steatotic liver disease.

Although the effects of smoking on human physiology are generally well understood, including increased exposure to DNA adduct-inducing nitrosamines, vascular damage from tar, and heavy metal accumulation through well-established pathways, this understanding does not extend to PSC, where such mechanisms remain poorly defined [9]. The role of smoking in PSC is complex and somewhat paradoxical, particularly given the close association between PSC and IBD. Smoking is well known to exacerbate Crohn’s disease (CD) but appears to have a protective effect in UC, which has a strong association with PSC [10]. From a mechanistic perspective, studies have shown that smoking exerts both direct and indirect effects on various factors, including oxidative stress, disruption of the intestinal barrier, immune cell dysfunction, as well as epigenetic alterations and changes in microbiota composition. These mechanisms may contribute to the complex relationship between smoking and IBD, and by extension, PSC (Figure 1). However, a direct link between smoking and PSC remains unestablished.

A case-control study by Mitchell et al. [11] examined the smoking habits of 170 patients with PSC, of which 41 without IBD, 170 patients with UC but normal liver function tests, and 170 age- and sex-matched community controls. A subanalysis of this study found that only 12 patients with PSC (7%) were current smokers, compared with 43 controls (25%). Additionally, 112 patients with PSC (66%) had never smoked, compared with 66 controls (39%). The odds ratio (OR) of having PSC was 0.17 [95% confidence interval (CI): 0.08, 0.35] among current smokers and 0.33 (95% CI: 0.21, 0.52) among ever (former + current) smokers. These results suggest that smoking may have a protective effect against the development of PSC, irrespective of the presence of IBD when comparing patients with PSC with and without IBD. Similarly, a population-based case-control study in the Netherlands involving 343 patients with PSC, 370 IBD controls, and 232 healthy controls found that smoking was associated with a lower risk of developing PSC in both patients with PSC-UC and PSC-CD [12]. The adjusted ORs were 0.21 (95% CI: 0.12, 0.34) for patients with PSC-UC and 0.17 (95% CI: 0.08, 0.39) for PSC-CD, compared with IBD controls. These findings further support the hypothesis that smoking may reduce the risk of PSC, independent of its protective effect against IBD [12]. In apparent agreement, a comprehensive assessment of environmental exposures among 1000 North American patients with PSC revealed that smoking was associated with PSC only when IBD was present (OR: 0.5; 95% CI: 0.4, 0.7), but not among those patients with PSC without IBD (OR: 0.9; 95% CI: 0.7, 1.2) [13], whereas additionally a systematic review and meta-analysis of 7 case-control studies, encompassing over 2 million participants, demonstrated that current smokers had a significantly lower risk of PSC compared with nonsmokers, with a pooled OR of 0.31 (95% CI: 0.18, 0.53). Former smokers also exhibited a reduced risk (OR: 0.52; 95% CI: 0.44, 0.61). Notably, the decreased risk persisted even when analyses were restricted to patients with PSC without IBD [14]. Finally, ever daily smoking before the diagnosis of PSC was associated with older age at diagnosis compared with never daily smoking (median 42 compared with 32 y; *P* < 0.001) [15]. Overall, the evidence that smoking influences the natural course of PSC appears compelling (Figure 1).

Mechanistically, it has been hypothesized that nicotine and other tobacco components could influence immune pathways relevant to PSC pathogenesis, potentially through modulation of T-cell responses or cytokine profiles [16,17]. Interestingly, the α4β2 nicotinic acetylcholine receptor augments intestinal macrophage phagocytosis [18]. Reduced macrophage activity has been linked to IBD in general [19,20] and is reported to be a causal factor in PSC in particular [21]. Hence, direct nicotine-mediated stimulation of macrophage action is a plausible mechanistic link here, and future studies in vaping individuals (which are exposed to mainly nicotine and not to many of the other bioactive compounds in tobacco smoke) may provide support for this hypothesis. An additional pathway is modulation of the gut microbiome. Smoking is well known to affect the microbiome and has been associated with increased colonization of the intestine with Shigella [22]. We ourselves observed that Shigella colonization of the colon is associated with a strongly reduced risk for recurrence of disease following liver transplantation for PSC [23]. Nonetheless, these mechanisms remain speculative, and in lieu of clear-cut mechanistic insight on how smoking influences the natural course of disease in PSC, the harmful effects of smoking on overall health strongly outweigh any uncertain potential benefit in PSC.

### Alcohol consumption

The impact of alcohol intake on PSC remains inconclusive. Given the hepatotoxic potential of alcohol, consumption is generally discouraged in patients with chronic liver disease, including PSC. Nevertheless, investigations into moderate alcohol use have raised questions about its potential role in autoimmune and inflammatory disorders as a contributing factor for PSC onset.

A retrospective study by Hagstrom et al. [24] evaluated the association between alcohol consumption and PSC outcomes. This study reported that moderate alcohol intake was not associated with an increased risk of disease progression; some biochemical markers (e.g., liver enzyme levels) tended to be more favorable among moderate drinkers, although these differences did not reach statistical significance. Andersen et al. [15] examined multiple lifestyle factors, including alcohol use, in relation to PSC risk. Their analysis indicated that moderate alcohol consumption did not significantly alter the risk of developing PSC, even though other factors such as coffee consumption and smoking appeared to show stronger associations. Furthermore, Lindqvist et al. explored the potential immunomodulatory effects of alcohol in patients with PSC, suggesting that while heavy alcohol use is clearly harmful, moderate consumption might exert subtle anti-inflammatory effects through modulation of immune pathways [25,26].

**FIGURE 2:**
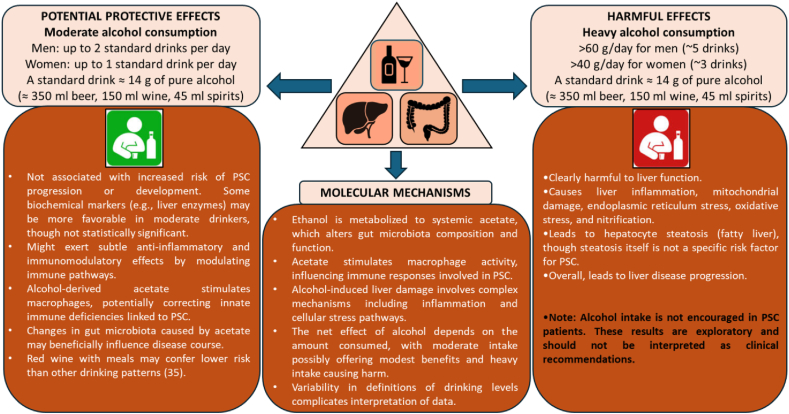
Hypothesized mechanisms linking alcohol to primary sclerosing cholangitis (PSC).

The definition of “moderate” alcohol intake varies across studies and countries [27]. For example, moderate consumption is often defined as 1–2 drinks per day for men and 1 drink per day for women in the United States, whereas other countries may set different thresholds [[27], [28], [29], [30]]. Most studies did not specify the type of alcoholic beverage, but national drinking patterns suggest that the majority of moderate alcohol consumption in these cohorts was likely through drinking beer or wine [[31], [32], [33]]. Importantly, fermented alcoholic beverages may contain bioactive compounds, such as xanthohumol in hops and resveratrol in red wine, which have anti-inflammatory properties even at low doses [31,32,34,35] (Figure 2).

Mechanistically, alcohol and its metabolites may interact with PSC pathophysiology in multiple ways. Alcohol metabolism produces acetate and acetaldehyde, which can modulate gut microbiota composition and function [36]. Martino et al. [36] demonstrated that acetate, rather than ethanol itself, drives microbiome changes associated with alcohol-related liver disease. These microbiome alterations may influence PSC, given the established connection between gut microbiota and disease course. Acetate may also stimulate macrophage activity, potentially benefiting PSC by compensating for innate immune deficiencies [37,38] (Figure 2). Furthermore, moderate alcohol intake has been associated with altered adiponectin expression in adipose tissue, which may contribute to systemic anti-inflammatory effects [39,40]. Bin Gao’s recent studies highlight the role of acetaldehyde in modulating immune responses, suggesting an additional mechanistic pathway for alcohol’s impact on inflammation [41,42].

However, alcohol-induced liver injury remains complex, involving inflammation, mitochondrial dysfunction, endoplasmic reticulum stress, nitrification, and oxidative stress [43,44]. The relevance of these processes in the context of moderate alcohol intake is not fully understood and appears largely related to hepatocyte steatosis, which is not considered a specific risk factor for PSC [45,46].

In summary, although limited data suggest that moderate alcohol consumption may not worsen and could potentially modestly benefit the clinical course of PSC, these findings are preliminary [47]. Mechanistic studies, including those focusing on acetate, acetaldehyde, adiponectin, and bioactive compounds in fermented beverages, provide hypotheses for potential effects, but overall evidence remains inconsistent and requires further research [33,40,48].

### Physical activity and sedentary behavior

Physical activity plays a crucial role in modulating immune function and reducing systemic inflammation. Regular moderate exercise enhances immunosurveillance by increasing the circulation of natural killer cells and cytotoxic T lymphocytes, thereby strengthening the body’s defense mechanisms against pathogens and potentially mitigating autoimmune responses. Additionally, consistent physical activity has been shown to lower levels of proinflammatory cytokines, contributing to improved immune regulation [49].

In the context of liver diseases, including autoimmune and metabolic disorders, physical activity has demonstrated beneficial effects. For example, in nonalcoholic fatty liver disease (NAFLD), increased physical activity correlates with reduced hepatic fat accumulation and improved liver function tests. Although specific studies on PSC are limited, the positive impact of exercise on liver health suggests potential benefits for patients with PSC as well [50].

Conversely, sedentary behavior is associated with adverse health outcomes, including increased inflammation and higher risk of developing liver diseases [51]. Prolonged sitting time has been linked to elevated markers of liver dysfunction, independent of physical activity levels [52]. This underscores the importance of not only engaging in regular exercise but also minimizing sedentary periods throughout the day.

Given these considerations, incorporating regular physical activity and reducing sedentary behavior may offer therapeutic benefits for individuals with PSC [53]. However, further research is necessary to establish specific guidelines and to understand the extent of these benefits in the PSC population.

## Surgical and Medical History Factors

### Appendectomy

The appendix was once considered a vestigial organ, but this view has changed since the early 2000s. Research suggests that the appendix may serve as a reservoir for beneficial gut bacteria through the release of biofilms [54]. Appendectomy has been extensively investigated in the context of UC, a disease that coexists with PSC in ≤80% of cases. Several studies have reported significantly lower rates of appendectomy in patients withUC compared with healthy controls, supporting the hypothesis that the appendix plays an important immunomodulatory role inUC. A systematic review including 6 observational studies comprising 5 case-control studies and 1 cohort study with a total of 2532 patients, was unable to conduct a meta-analysis due to clinical heterogeneity [55]. Among these studies, 1 demonstrated that patients who underwent appendectomy before UC onset experienced significantly lower relapse rates [absolute risk reduction (ARR) = 21.5%; 95% CI: 1.71%, 45.92%]. Two studies reported a reduced need for immunosuppressive therapy in patients after appendectomy (ARR = 20.2%; 95% CI: 9.67%, 30.46% and ARR = 21.4%; 95% CI: 10.32%, 32.97%, respectively) [56,57].

In the context of IBD, a colectomy refers to the surgical removal of all or part of the colon. It is most commonly performed in patients with UC**,** and less frequently in CD, depending on the severity, complications, or failure of medical therapy [58]. Colectomy rates showed mixed results, with 1 study indicating higher colectomy rates among patients without appendectomy (ARR = 8.7%; 95% CI: 1.29%, 18.66%) [58], whereas 2 others observed lower colectomy rates in patients after appendectomy (ARR = 21.4%; 95% CI: 13.17%, 28.79% and ARR = 18.7%; 95% CI: 7.50%, 29.97%) [56,57]. Overall, the current body of biomedical literature supports the hypothesis of an immunological role for the appendix in regulating gut immune responses and maintaining immune tolerance, possibly through modulation of mucosal immunity or gut microbiota.

The potential impact of appendectomy on PSC has been investigated in a limited number of studies, and with somewhat conflicting results.

Mitchell et al. [11] conducted a Swedish cohort study and observed that patients with PSC with a history of appendectomy experienced a significantly delayed onset of PSC compared with those without appendectomy. This delay in disease onset was accompanied by a milder clinical phenotype, including less aggressive IBD requiring fewer colectomies, suggesting that appendectomy may modulate disease expression both in PSC and associated IBD.

Further supporting the hypothesis of a protective role of appendectomy, Andersen et al. [15] reported that appendectomy was less frequent among patients with PSC with IBD compared with patients with IBD without PSC. This differential appendectomy rate implies that removal of the appendix might reduce the likelihood of PSC development in individuals with underlying bowel inflammation, possibly by altering immune pathways or antigen presentation that contribute to liver inflammation and fibrosis in PSC. However, other findings have been reported as well.

In a large multicenter study, Eaton et al. [13] found no significant association between appendectomy and the risk of developing PSC or differences in disease severity and progression. Similarly, Boonstra et al. [12] evaluated appendectomy status in a large cohort of patients with PSC and did not find meaningful effects on disease progression, transplant-free survival, or clinical outcomes. Both studies acknowledged that confounding by the presence and activity of IBD poses a significant challenge in interpreting the relationship between appendectomy and PSC.

Mechanistically, appendectomy produces variable but distinct effects on the composition of the gut microbiota. Among the prominent effects of appendectomy on the microbiome is an upregulation of colon adherent *Ruminococcus* [59] and downregulation of *Burkholderiaceae* levels. Intriguingly, patients with PSC have lower fecal *Ruminococcus* levels [60], whereas *Ruminococcus* is an important luminal producer of acetate (which may have anti-PSC activity, as discussed in paragraph 2.2) and oral gavage with *Ruminococcus* of mice with experimental fibrosis counteracted disease progression [61]. Therefore, it would be interesting to investigate the potential of *Ruminoccus* species to prevent the development of PSC in patients with UC or to modify the natural history of disease in patients with PSC. The feasibility of this approach has been demonstrated in IBD, where a *Lactococcus lactis* strain expressing the immunosuppressive cytokine IL-10 has shown promising results in clinical studies [62]. IL-10 is a key anti-inflammatory cytokine that suppresses the production of proinflammatory mediators, reduces activation of immune cells, and protects tissues from damage caused by chronic inflammation. Due to these properties, IL-10 helps decrease inflammation and promotes tissue regeneration, making it a promising therapeutic agent for inflammatory diseases. Therefore, a similar approach using genetically modified *Ruminococcus* could potentially be effective in treating IBD-associated PSC as well. Overall, although appendectomy may influence the microbiome environment and consequently the natural history of PSC, particularly in the context of coexisting IBD, the current evidence remains inconclusive regarding its impact on long-term disease course or prognosis. The appendix’s role as a reservoir of gut microbiota suggests a plausible mechanisms by which appendectomy could modify gut–liver immune interactions implicated in PSC pathogenesis. To clarify these associations, well-designed prospective studies that control for IBD status and activity are needed. Such research could not only elucidate the immunopathogenesis of PSC but also potentially inform therapeutic strategies targeting the gut–liver axis.

### Tonsillectomy

Tonsillectomy has been explored as a potential environmental and immunological modifier in the pathogenesis of PSC, given its established links with immune-mediated conditions such as UC, with which PSC frequently coexists [63]. The tonsils are lymphoid organs involved in mucosal immune surveillance and are thought to influence systemic immune responses through cytokine production and modulation of T-cell subsets, even in the absence of convincing evidence for tonsillectomy to significantly affect either humoral or cellular immunity [64].

A case-control study by Mitchell et al. [11] assessed the prevalence of prior tonsillectomy in 170 patients with PSC, 170 patients with UC without liver disease, and 170 healthy controls. The study found that only 21% of patients with PSC had undergone tonsillectomy, compared with 31% of healthy controls, suggesting a possible protective effect. However, this difference reached statistical significance (*P =* 0.05), but no significant association was identified between tonsillectomy and UC status [11].

A large North American cohort study involving over 1000 patients with PSC also found no significant association between tonsillectomy and PSC risk. The reported OR for PSC in individuals with a history of tonsillectomy was 0.9 (95% CI: 0.7, 1.1; *P =* 0.23), indicating no clear protective or predisposing effect [13]. Taken together, these data suggest that the role of tonsillectomy in PSC remains inconclusive and likely minimal. At present, there is insufficient evidence to support tonsillectomy as a preventive or therapeutic intervention for either condition outside standard indications.

### Cholecystectomy

In the context of PSC, cholecystectomy holds additional clinical significance and complexity. PSC is frequently associated with biliary abnormalities that increase the risk for gallbladder malignancy, making gallbladder surveillance a key component of disease management [65].

Several studies have shown that gallbladder polyps and masses are more common in patients with PSC than in the general population, and when present, they are significantly more likely to be malignant. In a large retrospective cohort, gallbladder masses were identified in ∼13.7% of patients with PSC undergoing cholecystectomy, with malignancy confirmed in 57% of these cases. These findings underscore the importance of timely cholecystectomy for suspicious lesions in patients with PSC, as delays may increase the risk of cholangiocarcinoma or gallbladder carcinoma [66].

Despite its potential therapeutic value, cholecystectomy in PSC is not without risks. A recent population-based study performed by Miyake et al. [67] reported that prior cholecystectomy in patients with PSC was associated with indicators of more severe liver disease, including higher Mayo risk scores and increased Fibrosis-4 Index (FIB-4) fibrosis indices. This suggests a potential link between cholecystectomy and accelerated disease progression, although causality remains uncertain.

Additionally, patients with PSC, particularly those with cirrhosis or portal hypertension, face a higher risk of perioperative complications. Postoperative morbidity is significantly elevated in this population, and careful preoperative risk stratification is essential. According to a study by Eaton et al. [68], patients with PSC undergoing cholecystectomy had higher rates of bile duct injury and infectious complications compared with non-PSC controls.

Importantly, emerging data suggest that the gallbladder may play a protective role in PSC pathophysiology. A recent study by Cazzagon et al. [66] demonstrated, in both human and mouse models, that the presence of the gallbladder may mitigate bile duct injury and inflammation. In murine PSC models, gallbladder removal exacerbated biliary injury, whereas gallbladder preservation appeared to support mucosal protection and bile flow regulation. These findings suggest that, beyond its role in storing bile, the gallbladder may contribute to maintaining immunological and functional balance in PSC, and its removal could potentially hasten disease progression [69].

Taken together, these data highlight the need for an individualized, risk-benefit approach when considering cholecystectomy in PSC.

### Hormonal therapy, pregnancy, and reproductive factors

Sex-based differences in autoimmune and cholestatic liver diseases suggest a role for hormonal and reproductive factors in disease susceptibility [70,71]. Although primary biliary cholangitis (PBC) shows a marked female predominance (10:1), PSC occurs more often in men (ratio: 2:1), despite IBD generally affecting women more frequently [72]. This inverse distribution raises questions about hormonal influences in PSC pathogenesis.

### Oral contraceptives and hormone replacement therapy

Several epidemiological studies have assessed exogenous hormones, including oral contraceptives (OCPs) and hormone replacement therapy (HRT), in PSC. Most large-scale analyses found no consistent association between OCP/HRT use and PSC risk or progression [13,73]. Some small studies suggested neutral or mildly protective effects, but data are limited by bias and confounding [20,62]. Unlike PBC, where hormonal factors are often implicated, PSC lacks strong evidence for such links [74]. Mechanistic studies, however, indicate that estrogens and progesterone influence bile acid metabolism, cholangiocyte proliferation, and immune signaling, supported by the presence of hormone receptors on cholangiocytes [[75], [76], [77]].

### Pregnancy and parity

Pregnancy introduces significant hormonal and immune changes, but evidence on PSC is sparse [78,79]. Available registry data suggest that pregnancy is generally well tolerated in stable PSC, though mild biochemical fluctuations and increased pruritus can occur [80,81]. One population study reported a higher risk of preterm birth (hazard ratio 3.6), emphasizing the need for high-risk obstetric care [82]. The effect of reproductive history including parity, menarche, or intrahepatic cholestasis of pregnancy on PSC progression remains unclear due to limited data.

## Dietary and Nutritional Factors

Diet plays a significant role in shaping immune function, metabolic homeostasis, and the composition of the gut microbiota, all of which are implicated in the pathogenesis of PSC [13,26, 83,84]. Although specific dietary guidelines for PSC are lacking, emerging evidence suggests that certain dietary components may influence disease risk, progression, or symptom burden.

### Coffee consumption

Among dietary exposures, coffee has consistently garnered attention in liver disease research. Numerous epidemiological studies have demonstrated an inverse association between regular coffee intake and the risk of chronic liver conditions, including NAFLD, cirrhosis, and hepatocellular carcinoma [15,85,86]. Coffee consumption is also linked to lower serum levels of liver enzymes such as γ-glutamyl transpeptidase and alanine aminotransferase (ALT), and a slower progression of liver fibrosis.

In the context of PSC, observational data suggest that coffee may have a protective role. Patients with PSC report significantly lower coffee intake compared with healthy controls. Andersen et al. [15] found that current consumption median 2.7 cups per day in patients with PSC compared with 4.0 cups in controls (*P <* 0.001), and at age 18, 1.0 compared with 1.6 cups, respectively (*P =* 0.001). Additionally, fewer patients with PSC consumed coffee daily both currently (76% compared with 86%; *P =* 0.006) and at age 18 (35% compared with 49%; *P =* 0.004).

Supporting these findings, Lammert et al. [85] reported that 24% of patients with PSC had never consumed coffee, compared with 16% of control subjects (*P <* 0.05), and only 67% of patients with PSC were current drinkers, compared with 77% of controls (*P <* 0.05). Lifetime coffee intake was also lower in patients with PSC (45 compared with 47 cups/mo; *P <* 0.05), and they had spent a smaller proportion of their lives drinking coffee (46.6% compared with 66.7%; *P <* 0.05). These associations remained statistically significant after multivariate adjustment. Notably, among patients with PSC with coexisting UC, coffee consumption was associated with a significantly lower risk of undergoing proctocolectomy (hazard ratio 0.34; *P <* 0.001).

A separate study further demonstrated that coffee consumption was associated not only with delayed disease progression in both alcoholic liver disease and PSC, but also with improved long-term survival following liver transplantation [87]. These findings suggest that regular coffee intake might be a beneficial, low-risk adjunctive recommendation in the clinical management of chronic liver diseases, including PSC [[88], [89], [90]].

Several biologically plausible mechanisms have been proposed to explain the protective associations of coffee with PSC and other chronic liver diseases, involving both caffeine-dependent and caffeine-independent pathways [91,92]. Caffeine acts primarily as an adenosine receptor antagonist, particularly at the A1 and A2A subtypes, counteracting the depressant effects of adenosine and enhancing acetylcholine release, an effect potentially relevant to PSC given the role of macrophage nicotinic acetylcholine receptors in its pathogenesis [93]. In addition, caffeine can increase cyclic AMP levels through nonselective phosphodiesterase inhibition and promote calcium release from intracellular stores, while also antagonizing gamma-aminobutyric acid receptors, although these latter effects generally occur at concentrations beyond typical human intake [[94], [95], [96]]. These mechanisms suggest that caffeine may influence immune signaling and hepatobiliary physiology beyond its central nervous system effects [95].

In contrast, caffeine-independent effects are largely mediated by other coffee components such as polyphenols, melanoidins, and additional secondary plant compounds [[97], [98], [99]]. These include anti-inflammatory activity through inhibition of proinflammatory cytokines like TNF-α, antioxidant effects, modulation of the gut microbiota, improvement of intestinal barrier integrity, stimulation of bile flow, and hepatocyte-protective signaling, which may reduce bile stasis and episodes of cholangitis [[100], [101], [102], [103], [104], [105]]. Experimental studies suggest that these bioactive compounds contribute significantly to the overall hepatoprotective effect of coffee beyond caffeine alone [106,107].

Experimental data support these mechanisms. In a preclinical study by Vitaglione et al. [86], rats fed a high-fat, high-calorie diet to induce steatohepatitis received 1.5 mL/d of decaffeinated coffee, polyphenols, or melanoidins for 8 wk equivalent to ∼2 cups of filtered coffee or 6 espressos per day in humans. Compared with high-fat diet controls, treated groups exhibited reductions in hepatic fat and collagen deposition, improved serum ALT and triglyceride profiles, and enhanced antioxidant status, as indicated by a lower oxidized/reduced glutathione ratio, decreased malondialdehyde levels, and increased ferric reducing antioxidant power [108]. Furthermore, coffee consumption downregulated proinflammatory and profibrotic mediators such as TNF-α, interferon gamma, tissue transglutaminase, and transforming growth factor β, while upregulating anti-inflammatory cytokines including IL-4 and IL-10, along with increased expression of the adiponectin receptor and peroxisome proliferator-activated receptor α in liver tissue [109,110].

Although causality cannot be inferred from observational data alone, the convergence of epidemiological and experimental evidence supports a biologically plausible hepatoprotective role for coffee in PSC and other chronic liver diseases. These findings underscore the potential importance of secondary plant compounds in mediating protective effects and highlight the need for prospective cohort studies and mechanistic clinical trials to clarify the extent and pathways of these effects.

### Vitamin intake

Several vitamins with antioxidant, immunomodulatory, and metabolic effects have been investigated in autoimmune liver diseases, including PSC [111]. However, the evidence specific to PSC remains relatively limited and often extrapolated from studies in broader cholestatic or chronic liver diseases.

#### Vitamin D

Vitamin D is of particular interest due to its critical immunoregulatory functions. Deficiency of vitamin D is highly prevalent among patients with PSC, likely resulting from impaired absorption and altered metabolism associated with cholestasis [112,113]. Low serum levels of 25-hydroxyvitamin D [25(OH)D] have been correlated with more advanced liver fibrosis and a greater risk of liver-related complications [26,114]. Vitamin D modulates both innate and adaptive immune responses, which may influence the autoimmune mechanisms implicated in PSC pathogenesis [115,116]. Despite these associations, interventional trials assessing the effects of vitamin D supplementation on PSC outcomes are lacking, and its clinical benefit remains to be determined.

#### Fat-soluble vitamins A and E

Fat-soluble vitamins A and E are also frequently deficient in cholestatic liver diseases due to impaired bile salt-dependent intestinal absorption. Vitamin A deficiency can contribute to impaired vision and immune dysfunction, whereas vitamin E, a potent antioxidant, may counteract oxidative stress implicated in liver injury [117,118]. Although vitamin E supplementation has demonstrated therapeutic benefit in other chronic liver conditions such as nonalcoholic steatohepatitis [118], evidence supporting its use in PSC is anecdotal and insufficient to recommend routine supplementation.

#### Vitamin K

Vitamin K deficiency is common in advanced liver disease and is primarily associated with coagulopathy due to impaired synthesis of clotting factors [119]. Although correction of vitamin K deficiency is crucial to prevent bleeding complications, there is no current evidence that vitamin K status influences the progression of PSC specifically [120].

#### Vitamin B6

Vitamin B6 deficiency, indicated by low pyridoxal 5'-phosphate (PLP) levels, is common in PSC, affecting 17%–38% of patients across multiple cohorts [121]. This deficiency is more pronounced in PSC than in IBD without PSC or PBC and is linked to disruptions in PLP-dependent metabolic pathways. Notably, low PLP levels often persist after liver transplantation and independently predict reduced transplantation-free survival in both pretransplant and posttransplant patients with PSC.

These findings suggest that vitamin B6 deficiency plays a role in disease progression rather than being a simple consequence of liver dysfunction [114,122]. Given the reduced microbial capacity to produce essential nutrients in PSC, routine monitoring of vitamin B6 status is warranted. Supplementation or modulation of gut microbiota may represent promising strategies to improve outcomes in PSC.

Given these considerations, routine monitoring of fat-soluble vitamin levels A, D, E, and K, and appropriate supplementation when deficiencies are detected, are standard components of managing patients with advanced cholestatic liver disease [123]. This approach may also be applicable in earlier stages of PSC to prevent complications related to vitamin deficiencies and potentially modulate disease progression [124]. However, further research, including prospective clinical trials, is needed to clarify the impact of vitamin supplementation on PSC outcomes and to establish evidence-based guidelines.

### Probiotics and gut microbiota

The gut microbiome plays a critical role in the pathogenesis of PSC, particularly given the strong association between PSC and IBD and the impact of gut-derived bacterial products on hepatic immune activation [60,125]. Dysbiosis, characterized by reduced diversity of beneficial bacteria and an increase in pathogenic species, has been consistently observed in patients with PSC [126,127].

Diet exerts a profound influence on the gut microbiota, and dietary interventions may offer therapeutic potential in PSC. High fiber diets promote the growth of beneficial bacteria such as *Bifidobacteria* and *Lactobacilli*, which produce short-chain fatty acids (SCFAs) like butyrate [128]. SCFAs support intestinal barrier integrity, regulate immune responses, and mitigate systemic inflammation, mechanisms that could contribute to improved liver health in PSC [129].

Probiotics have demonstrated promise in small clinical trials, suggesting their ability to restore microbiome balance, reduce endotoxin translocation, and improve liver biochemistry in some patients with PSC [130,131]. However, results have been inconsistent, and more rigorous studies are needed to identify effective strains and dosing strategies [132]. Clinical studies to date most frequently investigated *Lactobacillus rhamnosus GG* and *Bifidobacterium longum*, as well as mixed formulations containing both *Lactobacilli* and *Bifidobacteria* [[133], [134], [135]]. In some cases, *Saccharomyces boulardii* was tested, though evidence in PSC remains very limited [136,137].

Additional dietary components such as omega (ω)-3 (n−3) fatty acids, polyphenols, and prebiotics, for example, nondigestible fibers such as inulin and resistant starch also influence gut microbiota composition and growth and may modulate inflammatory signaling along the gut–liver axis [[138], [139], [140], [141]], potentially by increasing SCFA production [142]. Although these compounds have shown beneficial effects in other chronic liver diseases, evidence specific to PSC remains limited and preliminary.

Probiotic strains like *Lactobacillus* and *Bifidobacterium* have been specifically studied in PSC contexts, with some small trials reporting reductions in alkaline phosphatase and improvements in gut barrier function by decreasing intestinal permeability [84,131,143]. Nonetheless, larger randomized controlled trials are necessary to confirm these findings and formulate clinical guidelines.

Dietary fiber, particularly fermentable fibers that are metabolized by the gut microbiota into SCFAs, plays a key role in maintaining intestinal barrier integrity and modulating immune responses. In IBD, which frequently coexists with PSC, high fiber intake has been associated with reduced disease activity and improved gut health [[144], [145], [146]]. Though direct evidence in PSC remains limited, mechanistic rationale exists. Among the fiber types studied, inulin, resistant starch, and β-glucan have been associated with increased butyrate production and the promotion of beneficial microbial populations such as *Bifidobacteria* and *Faecalibacterium prausnitzii* [[147], [148], [149]]. Psyllium has also shown beneficial effects in IBD, which may be relevant to PSC [150,151]. SCFAs produced by microbial fermentation of fibers can reduce systemic and hepatic inflammation, enhance gut–liver axis communication, and inhibit proinflammatory signaling pathways [152,153]. Higher fiber intake may also promote beneficial microbial populations, reduce gut-derived endotoxemia, and attenuate hepatic immune activation all of which are relevant in PSC pathogenesis [154,155].

### Polyunsaturated fatty acids

ω-3 PUFAs, such as EPA and DHA, possess anti-inflammatory properties and have been explored in various chronic liver diseases [156,157]. In autoimmune liver conditions, ω-3 PUFAs may help modulate immune responses and suppress hepatic inflammation through activation of resolution pathways. These pathways include specialized pro-resolving mediators such as resolvins, protectins, and maresins, which promote the cessation of neutrophil infiltration, enhance clearance of apoptotic cells, and reduce proinflammatory cytokine production. Additionally, ω-3 PUFAs inhibit activation of the nuclear factor kappa-light-chain-enhancer of activated B cells (NF-κB) signaling pathway, modulate T-cell profiles toward regulatory phenotypes, activate the G protein-coupled receptor 120 (GPR120) receptor that suppresses macrophage activation, and stimulate production of anti-inflammatory cytokines like IL-10. Together, these mechanisms contribute to reducing inflammation and maintaining immune homeostasis in autoimmune liver diseases [158]. Although clinical trials specifically evaluating PUFAs in PSC are sparse, some evidence suggests they may ameliorate bile duct injury and reduce hepatic fibrogenesis [159]. However, data are currently insufficient to support routine supplementation in patients with PSC. Ongoing research in related liver diseases may help clarify their therapeutic potential.

In conclusion, PSC is a complex, immune-mediated cholangiopathy with no established cure and limited treatment options. Its pathogenesis is strongly influenced by genetic predisposition and the presence of IBD, but accumulating evidence suggests that modifiable environmental, dietary, and lifestyle factors may also play a role in disease onset, progression, or phenotype.

Among factors with more consistent observational or mechanistic evidence, moderate coffee consumption and adequate vitamin status appear to be associated with potential hepatoprotective effects, and their modification may be considered relatively safe within general dietary guidelines. Surgical history, such as prior colectomy in the setting of IBD, also shows associations with PSC outcomes but is not modifiable.

Other associations remain preliminary or speculative, including the impact of smoking, hormonal exposures, probiotics, dietary fiber, and fermented alcoholic beverages (Table 1). These factors require cautious interpretation due to confounding by IBD, small sample sizes, and retrospective designs.

**TABLE 1:** Lifestyle, environmental, and nutritional factors in primary sclerosing cholangitis: evidence summary, mechanisms, and clinical implications.

Factor	Observations in PSC	Proposed mechanisms	Clinical implications	Source
Smoking	Lower prevalence in PSC; reduced odds with current/ever smoking; no difference in IBD vs. non-IBD subgroups; later initiation in PSC	Immune modulation via nicotine; altered T-cell responses; speculative	Appears protective but not recommended clinically due to health risks; further study needed	[[16], [17], [18], [19], [20]]
Alcohol consumption	Moderate intake not linked to progression; possibly better biochemistry but not significant; heavy use harmful	Subtle anti-inflammatory effects; immune modulation	Moderate use not harmful, but not recommended therapeutically	[20,29,30]
Physical activity	No PSC-specific data; general liver data show benefits; sedentary behavior worsens liver health	Enhances T/NK cells, ↓ cytokines, ↓ hepatic fat, immune modulation	Encourage regular exercise; supports general liver and immune health	[37,41]
Appendectomy	Mixed evidence; possibly delays PSC onset and milder disease in IBD; lower rates in PSC + IBD	Mucosal immunity and microbiota alteration; gut–liver axis involvement	Possibly protective; not actionable yet; may guide future research	[[16], [17], [18],^20^]
Tonsillectomy	No clear association with PSC or disease severity	Possible Th2→Th1 shift; immune modulation	No current clinical utility in PSC management	[16,18,51]
Cholecystectomy	Higher malignancy risk in gallbladder; linked to worse PSC progression	Gallbladder regulates bile flow and mucosal immunity	Considered for malignancy risk; may accelerate PSC needs surgical caution	[18,[53], [54], [55],^57^]
Hormonal and reproductive factors	No strong link with OCP/HRT; pregnancy well tolerated but risk of preterm birth	Sex hormones affect bile acids, cholangiocytes, and immune pathways	OCP/HRT not contraindicated; manage pregnancy as high risk; mechanisms unclear	[18,20,58,59,64,68,70,71]
Coffee consumption	Lower intake in PSC; inversely linked to severity and surgery risk	↓ TNF-α, ↑ bile flow, gut microbiota modulation, antioxidant effects	May slow progression; safe, low-risk adjunct	[20,73,75,76]
Vitamin D	Common deficiency; linked to fibrosis and complications	Innate/adaptive immune effects; reduces fibrosis	Recommend monitoring and supplementation; potential disease-modifying role	[30,94,96]
Vitamins A, E, K	Often deficient in cholestatic liver disease	A: immunity, vision; E: antioxidant; K: clotting factors	Supplement as needed; no PSC-specific therapeutic data	[[99], [100], [101]]
Vitamin B6 (PLP)	17%–38% deficient; associated with worse survival	PLP disruption affects key metabolic pathways	Monitor and correct; may indicate disease severity	[96,108]
Probiotics	Patients with PSC show dysbiosis; small studies show improved markers	Restores microbiome, ↓ endotoxemia, strengthens gut barrier	Promising but unproven; larger trials needed	[48,113,118]
Prebiotics	Role in PSC not well-studied; support SCFA-producing bacteria	↑ Butyrate → ↓ inflammation, ↑ gut barrier integrity	Potential role; evidence lacking for PSC-specific outcomes	[119]
Dietary fiber	Limited PSC-specific data; beneficial in IBD and mechanistically relevant	↑ SCFAs → immune modulation, reduced hepatic inflammation	Encourage fiber intake; likely beneficial via gut–liver axis	[120,121,123]
ω-3 PUFAs	Limited PSC-specific data; known liver anti-inflammatory effects	Resolving inflammation; immune modulation	Insufficient evidence for PSC use; under investigation	[127,129]

Abbreviations: IBD, inflammatory bowel disease; HRT, hormone replacement therapy; OCP, oral contraceptive pill; PLP, pyridoxal 5’-phosphate; PSC, primary sclerosing cholangitis; SCFA, short-chain fatty acid; Th, T helper cell; T/NK cells, T lymphocytes/natural killer cells.

Understanding and validating these associations through high-quality, prospective research could enable risk stratification, preventive strategies, and adjunctive interventions. Even modest improvements achieved through lifestyle and dietary modifications may significantly affect patient outcomes and quality of life in a disease currently defined by limited therapeutic options.

## Author contributions

The authors’ responsibilities were as follows – RAM: responsible for conceptualization, literature search, and writing – original draft; HPR: contributed to data interpretation and critical review; LJWvdL: involved in critical review, editing, and references; MPP: undertook final manuscript revision and supervision; and all authors: contributed substantially to the conception and design of the review, drafting the manuscript, reading, and approving the final version.

## Data availability

Data described in the manuscript will not be made available because this is a narrative review and does not involve original research or new datasets.

## Declaration of generative AI and AI-assisted technologies in the writing process

During the preparation of this work, the author(s) used ChatGPT to grammar correction, sentence restructuring, and readability improvement during manuscript preparation. After using this tool/service, the author(s) reviewed and edited the content as needed and take(s) full responsibility for the content of the publication.

## Funding

The authors reported no funding received for this study.

## Conflict of interest

The authors report no conflicts of interest.
